# Meeting report of the fourth annual Tri-Service Microbiome Consortium symposium

**DOI:** 10.1186/s40793-021-00384-z

**Published:** 2021-08-21

**Authors:** Michael S. Goodson, Robyn A. Barbato, J. Philip Karl, Karl Indest, Nancy Kelley-Loughnane, Robert Kokoska, Camilla Mauzy, Kenneth Racicot, Vanessa Varaljay, Jason Soares

**Affiliations:** 1 711th Human Performance Wing, Air Force Research Laboratory, Wright-Patterson AFB, Dayton, OH USA; 2 United States Army Engineer Research and Development Center – Cold Regions Research and Engineering Laboratory, Hanover, NH USA; 3 Military Nutrition Division, United States Army Research Institute of Environmental Medicine, Natick, MA USA; 4 United States Army Engineer Research and Development Center, Vicksburg, MS USA; 5 Materials and Manufacturing Directorate, Air Force Research Laboratory, Wright-Patterson AFB, Dayton, OH USA; 6 Physical Sciences Directorate, United States Army Research Laboratory – United States Army Research Office, Research Triangle Park, Durham, NC USA; 7 Soldier Effectiveness Directorate, United States Army Combat Capabilities Development Command Soldier Center, Natick, MA USA

**Keywords:** Microbiota, Environmental microbiome, Military, Human performance, Microbiome engineering, Polymicrobial communities, Biotechnology, Synthetic biology

## Abstract

The Tri-Service Microbiome Consortium (TSMC) was founded to enhance collaboration, coordination, and communication of microbiome research among U.S. Department of Defense (DoD) organizations. The annual TSMC symposium is designed to enable information sharing between DoD scientists and leaders in the field of microbiome science, thereby keeping DoD consortium members informed of the latest advances within the microbiome community and facilitating the development of new collaborative research opportunities. The 2020 annual symposium was held virtually on 24–25 September 2020. Presentations and discussions centered on microbiome-related topics within four broad thematic areas: (1) Enabling Technologies; (2) Microbiome for Health and Performance; (3) Environmental Microbiome; and (4) Microbiome Analysis and Discovery. This report summarizes the presentations and outcomes of the 4th annual TSMC symposium.

## Introduction

The Tri-Service Microbiome Consortium (TSMC) was founded in 2016 to enhance collaboration, coordination, and communication of microbiome research among U.S. Department of Defense (DoD) organizations and to facilitate resource, material and information sharing among consortium members. Towards those goals, and to discuss applications and implications of microbiome research, the TSMC hosts an annual symposium that includes subject matter experts from federal/state agencies, DoD-affiliates, academic institutions, and industry [1–4]. The 2020 symposium was held virtually on 24–25th September, with 181 registered attendees (72% DoD, 6% DoD-affiliated, 5% non-DoD government, 9% academia, 2% industry, 6% International). This represents a 39% increase in attendees compared to the in-person third annual TSMC symposium [4]. The annual TSMC symposium attendances have been growing by about 30% each year [1–4]. This larger increase may be a result of the easier accessibility of a virtual platform. Over the 2 day symposium, 31 speakers presented on microbiome-related topics within four broad thematic areas: (1) Enabling Technologies; (2) Microbiome for Health and Performance; (3) Environmental Microbiome; and (4) Microbiome Analysis and Discovery. Forty eight percent of the speakers were female and 24% of the speakers were from underrepresented minorities. This represents a 3% decrease and 11% increase, respectively, compared to the third annual TSMC symposium [4]. Seventeen speakers (10 early-career) were from DoD laboratories (Table 1), five were from non-DoD government laboratories, six were from academic institutions (one from the United States Air Force Academy), and one was from industry. In lieu of the poster session that would have occurred if the meeting had been in-person, two sessions of pre-recorded 10 min ‘lightning talks’ were included in the agenda followed by a live question and answer session paneled by the lightning talk presenters.

**Table 1:** Summary of presented research

Organization	Microbiome area	Topic
*DoD laboratories*		
*Air Force*		
AFRL	Health and performance	Exploring changes in the host gut microbiota during a controlled human infection model for *Campylobacter jejuni*
AFRL	Environmental	Microbiomes of military aircraft and vehicles and their connection to biocorrosion and biodeterioration
USAFA	Environmental	Per- and polyfluoroalkyl substances (PFAS)-degrading microorganism mining
*Army*		
DEVCOM ARL	Analysis and discovery	Metagenomics and genome-scale metabolic modeling of microbes sampled from corroded Army equipment
DEVCOM SC	Enabling technologies	In vitro fermentation as a complement to human studies to explore gut microbial composition and metabolic capacity*
ERDC-CRREL	Enabling technologies	Growing the dark web: melanized fungal structures as a biological wire
ERDC-CRREL	Environmental	Determination of the ecological process structuring microbial communities during permafrost that using a phylogenetic null modeling approach
ERDC-CRREL	Environmental	DRTSPORE: a model that predicts soil activity across the landscape*
ERDC-CRREL	Environmental	Changes in permafrost microbial community composition after thaw*
ERDC-CRREL	Environmental	Cataloguing psychrophilic and psychrotolerant bacteria*
ERDC-CRREL	Environmental	UV-resistant psychrotolerant microorganisms: understanding the role of pigmentation for survival*
WRAIR	Health and performance	Longitudinal shift in fecal microbiota associated with acute and delayed effects of PTSD-eliciting stress model
WRAIR	Health and performance	Impact of radiation dose and temporal delay on host-biome relationships and fecal metagenomics*
*Navy*		
NRL	Analysis and discovery	Multiomic analysis of the gut microbiome of US Navy divers
NRL	Environmental	Compositional variability and biodegradative potential of the plastisphere in aquatic environments*
*Government laboratories*		
LANL	Enabling technologies	Utilizing single cell genomics and gel microdroplets to advance public health, environmental impact, and security
MIT-LL	Enabling technologies	A generalizable method for engineering non-model microbes
USDA-ARS	Enabling technologies	The in vitro human gut microbiome system*
US EPA	Environmental	EPA activities related to indoor microbiome and human health*
VA-Rocky Mountain MIRECC	Health and performance	Evaluation of an immunomodulatory probiotic intervention for veterans with co-occurring mild TBI and PTSD: a pilot study*
*Academia*		
Brigham and Women’s Hospital	Health and performance	Bone loss with aging is independent of age-related gut microbiome dysbiosis in mice
MIT	Enabling technologies	Mining the chemical signals human-associated bacteria use to control their host^$^
UCSD	Analysis and discovery	Metabolomics tools to study the molecular impact of the microbiome^$^
UMASS-Amherst	Environmental	Soil microbial adaptation to chronic soil warming^$^
Weizmann Institute of Science	Health and performance	Host-microbiome interactions in health and disease^$^
*Industry*		
DuPont Nutrition and Biosciences	Analysis and discovery	Comparative microbiome study in lean vs. obese population: discovery of novel probiotics for metabolic health

*Lightning talk; ^$^Keynote; *AFRL* Air Force Research Laboratory; *ARL* Army Research Laboratory; *CFD* Combat Feeding Directorate; *CRREL* Cold Regions Research and Engineering Laboratory; *DEVCOM* Combat Capabilities Development Command; *DoD* US Department of Defense; *EPA* Environmental Protection Agency; *ERDC* US Army Engineer Research and Development Center; *LANL* Los Alamos National Laboratory; *LL* Lincoln Laboratory; *MIRECC* Mental Illness Research Education and Clinical Center; *MIT* Massachusetts Institute of Technology; *NRL* Naval Research Laboratory; *SC* Soldier Center; *UCSD* University of California, San Diego; *UMASS* University of Massachusetts; *USAFA* US Air Force Academy; *USDA-ARS* US Department of Agriculture – Agricultural Research Service; *VA* Department of Veterans Affairs; *WRAIR* Walter Reed Army Institute of Research

This report details those activities in an effort to foster potential collaboration across scientific communities. The meeting was also recorded and can be viewed here: www.ues.com/tsmc2020

### Opening remarks

The Fourth Annual TSMC meeting was originally scheduled to be hosted at the US Army Engineer Research and Development Center – Cold Regions Research and Engineering Laboratory, and so the virtual meeting was opened by their Director, Dr. Joseph Corriveau. Dr Corriveau extolled the virtues of the agenda and emphasized that this meeting aligns with the strategic objectives of the DoD in Biotechnology, especially in providing a forum for stimulating innovative collaborations between the DoD service laboratories. Some of these collaborative efforts were highlighted during the TSMC overview, provided by the TSMC Chair, Mr. Jason Soares (DEVCOM SC), and TSMC Co-Chair, Dr. Michael Goodson (AFRL). As well as the annual meeting, TSMC knowledge products include: meeting reports [1–4], reviews [5, 6] and topical meetings focusing on specific areas within Microbiome research. During the overview, Mr. Soares noted the TSMC’s position as a team within the Optimizing Warfighter Performance subarea of the DoD Biotechnology Community of Interest (CoI), while also noting that the TSMC’s reach spanned all of the subareas within the CoI. The impact of the TSMC on the Biotechnology CoI was echoed by Dr. Rajesh Naik (AFRL), who oriented the meeting to this new construct. CoI’s are multi-agency collaborative efforts to define the direction of research in the DoD. The Biotechnology CoI’s vision is to expand and accelerate biotechnology modernization, develop science and technology policy and guidelines, and transition products into the hands of Warfighters through engineering biology, engineering with biology, and developing biotechnology enablers. An essential component of this is to foster cross-service partnerships, exemplified by this meeting, and to enhance workforce development through education of the existing and future workforce. Taken together, these opening remarks framed the importance of DoD microbiome research within the broad context of developing innovative biotechnologies for maintaining and advancing multiple and diverse national security interests.

### Enabling technologies

The enabling technologies session was initiated by a Keynote presentation from Prof. Christopher Voigt (MIT). Prof. Voigt discussed how his research group mines microbial genomes to find pathways and gene clusters that produce chemicals of interest and then expresses them in heterologous hosts. He demonstrated their ‘sequence to molecule’ pipeline that they use to identify the 14,000 unexplored gene clusters in the human gut microbiome, how they reconstruct them with synthetic control, and then how they express these in *E. coli* to explore what is produced [7]. To date 66 molecules have been found using this method, including 53 novel compounds and many that are difficult to produce chemically. He concluded his presentation by describing methods to program commensal bacteria, including *B. thetaiotaomicron* [8, 9].

The three technical talks that followed focused on technological advances that are advancing our understanding and utilization of microbial consortia. Dr. James Comolli (MIT-Lincoln Laboratory) described methods to engineer endogenous microbes in situ by modifying microbes using mobile DNA elements and then applying these to a mixed microbial community. Whereas other have used broad host range mobile elements [10], Dr. Comolli is characterizing precise mobile elements to target specific bacteria by utilizing different combinations of distinct mobile elements and screening which *Bacteroides* strains take them up through conjugation.

Continuing the theme of utilizing microbial consortia, Mr. Robert Jones (ERDC-CRREL) discussed the conductive properties of melanized fungal biomasses. Mr. Jones described how melanin is produced in the cell membrane, and is then extruded extracellularly between the fungal cell membrane and cell wall to form a sheath around the fungus. It is thought that this has evolved to protect the fungus from radiation damage by scavenging free radicals. However, melanin is also an ion and electron charge carrier potentiating it is a living conductive structure that could connect biology to electric devices.

The final talk in this session focused on technology that aids in the understanding of microbes at the individual level. Dr. Armand Dichosa (Los Alamos National Laboratory) discussed how single cell genomics (SCG) allows the ‘unknowns’: the novel, recalcitrant, and underrepresented bacteria to be investigated. SCG uses flow cytometry to isolate single cells from any microbial consortium, and employs the rolling circle technique to amplify the genome. Coupling SCG technology with gel microdroplet (GMD) techniques has allowed previously unculturable bacteria to be investigated [11, 12]. Briefly, GMD randomly captures single cells that can be combined to grow in a defined low cell number mixed community. Dr. Dichosa discussed how this co-culturing technique promotes cell growth by providing signals and substrates that would not be generated if cells were present in a monoculture. The ability to culture for weeks to months also enables slow growing organisms to be cultured. Dr. Dichosa closed by giving examples of where these technologies are being successfully applied, including isolating bacteria from an Antarctic tunicate whose genome contained a biosynthetic pathway for an anti-tumor compound, the ability to downselect organisms from the skin and gut microbiota that can suppress pathogen growth, and the identification of denitrifying bacteria capable of degrading aromatic compounds from sludge.

### Microbiomes for health and performance

Prof. Eran Elinav, Weizmann Institute of Science, Israel, was the keynote speaker for the microbiome for health and performance session. Prof. Elinav described the tools used for mechanistic understanding of microbiomes to avoid a ‘one-size fit all’ microbiome treatment approach. He then highlighted the ways in which his lab is developing microbiome treatments for health. Since diet is the dominant modulator of the gut microbiome, nutrition is a potential avenue to effect health benefits from the gut microbiome. However, there is high variability in the response to dietary changes across populations. To investigate this, Prof. Elinav’s research group initiated the ‘personalize nutrition project’ where subjects were monitored for diet, sleep, glucose levels, gut microbiome composition, blood metabolites, and mood. Using these data, individualized response to certain foods could be predicted, and when personalized diets were prescribed to 200 pre-diabetic individuals, their outcomes were improved compared to comparable diets suggested by current guidelines [13]. Microbiome transplants were then discussed, focusing on vaginal microbiome transplants. Bacterial vaginosis affects a third to a half of all women, and in around 10% of cases it is intractable and recurrent. However, transfer of the vaginal microbiome from a healthy control resulted in a full cure of refractory patients for greater than 3 years [14]. Refractory bacterial vaginosis microbiomes can be identified through sequencing, recognizing those patients that would benefit the most from a vaginal microbiome transplant, which is the basis for an on-going multinational study. Post-biotics, microbiome-produced small molecules and metabolites, were discussed next in the context of amyotrophic lateral sclerosis (ALS). ALS is very variable in terms of progression and manifestation, and the gut microbiome has a role in this variability. In mice ALS models, broad spectrum antibiotics that depleted the microbiome made the symptoms more severe. Studies showed that one commensal microbe was inversely correlated with disease progression, i.e., when that microbe was added to the ALS mouse model, the symptoms were reduced. Metabolomic profiling of that microbial species indicated that it produced nicotinamide, and when this small molecule was added to the ALS mouse model, symptoms were reduced. These data correlate with human fecal metabolome studies that showed that ALS patients had reduced nicotinamide levels compared to non-ALS controls that shared the same household, indicating that the gut microbiome can provide key metabolites in human interventions. Finally, the effect of the microbiome on host cells was discussed using an acute liver failure mouse model induced by acetaminophen intoxication. Deep microbiome characterization and single cell transcriptomics identified 82 genes that were differentially expressed in damaged liver cells compared to healthy liver cells. All of these genes were regulated by the transcription factor MYC, suggesting that MYC is a master regulator of acute liver failure. Microbiome depletion attenuated the acute liver failure, suggesting that factors produced by the microbiome regulates the MYC cascade [15]. Prof. Elinav concluded by reiterating that future treatment of diseases should consider the role of the microbiome.

The technical presentations within the microbiome for health and performance session included three talks that separately explored the dynamic relationships between the gut microbiota and the brain, bone, and gastrointestinal tract. Dr. Allison Hoke (Walter Reed Army Institute of Research) focused on a connection between the brain and gut microbiota by describing changes in fecal microbiota composition in response to a “cage-within-cage resident-intruder” model of psychological stress shown to elicit post-traumatic stress disorder- (PTSD) like traits in mice [16]. PTSD is a sometimes debilitating disorder that disproportionately affects military veterans, and which has several comorbidities linked to the gut microbiome. Study findings demonstrated that stress exposure altered fecal microbiota community composition, with the most notable effects being changes in diversity and relative abundance of Actinobacteria and Verrucomicrobia. Those results raised the intriguing question of whether stress-induced changes in the gut microbiome may contribute to the psychological and physiologic comorbidities of PTSD. In the future, confirming a link between the gut microbiome, PTSD and associated comorbidities could facilitate the development of novel gut-microbiome targeted therapeutics.

Dr. Xiaomeng You (Brigham and Women’s Hospital) explored relations between age-related gut microbiome dysbiosis and age-related bone loss. She presented results of several studies that used a combination of mouse models including aged mice, germ free and specific pathogen-free mice, and fecal microbiota transplant experiments. The gut microbiome is known to play a role in bone homeostasis via modulation of immune, growth and bacterial factors that directly and indirectly interact with pathways regulating bone remodeling [17]. Further, both gut microbiota and bone health are known to decline with age. However, results of the presented experiments did not demonstrate a causal role of dysbiosis in age-related bone loss. Musculoskeletal injuries, and stress fracture in particular, are common in military populations. While these findings suggest that the gut microbiome may not play a critical role in age-related bone loss, the established connection between the gut microbiome and bone health does suggest a potential role for the gut microbiome in military relevant bone health outcomes that warrants further exploration.

Travelers’ diarrhea is a leading cause of non-battle injury in deployed warfighters and identifying strategies to prevent TD is a top military priority [18, 19]. *Campylobater jejuni* is one of several TD-causing pathogens, especially in Southeast Asia. Dr. Blake Stamps (Air Force Research Laboratory) presented findings from a controlled human infection model of *C. jejuni* which explored whether the gut microbiota may explain inter-individual variability in susceptibility to and recovery from campylobacteriosis. Results from this preliminary study identified several commensal taxa that were associated with diarrhea severity, supporting the prospect that certain gut microbiota taxa or communities may confer protection against *C. jejuni* infection and potentially other TD-causing pathogens. The study also demonstrated the utility of mining data from controlled human infection model studies to identify novel taxa or communities that may protect against TD. Those microbes can then be validated in large cohort studies, and possibly facilitate development of novel probiotics for TD prevention.

### Environmental microbiome

The environmental microbiome session included an invited talk and three topical presentations discussing microbial communities found in frozen soils, contaminated soils, and aircraft vehicles. Dr. Kristen DeAngelis (University of Massachusetts-Amherst) discussed the effects of climate change on carbon dynamics, carbon storage, and greenhouse gas emissions in the context of plant–microbe interactions. Dr. DeAngelis uses the Harvard Forest, a deciduous forest in Massachusetts, to study the short-term and long-term effects of warming on carbon cycling in temperate forest soils [20]. Soil organic matter was not only reduced in the soil after 20 years of warming, but its composition had changed [20] with phases of soil carbon loss alternating with phases of no detectable loss of soil carbon [21]. In fact, soil microbial community structure adapted to changes in the composition of soil organic matter, particularly the increase in lignin [22] and a deeper investigation of the microflora using high throughput sequencing technologies show that the long-term soil warming is resulting in a different niche space for bacteria and fungi [23]. This work highlights the importance of studying long-term trends in climate change and its effect on nutrient cycling mediated by soil microorganisms.

Ms. Stacey Doherty (ERDC-CRREL) expanded on this topic to the effects of climate change on frozen terrain through investigation of ecological processes structuring microbial communities during permafrost thaw in Abisko, Sweden [24]. Permafrost is ground that has been frozen for at least two consecutive years and is a reservoir for carbon. A significant research gap is understanding how microorganisms will assemble to mediate this carbon once the permafrost thaws. The study used phylogenetic null modeling to determine microbial community assembly during permafrost thaw. A major finding was that stochastic (i.e. random) assembly was the predominant ecological process immediately following thaw. Then, deterministic (i.e. environmentally influenced) assembly became the main ecological process. The combinations of microorganisms assembling post-thaw will influence nutrient cycling and available metabolites for higher-order organisms [25].

Emerging contaminants such as per- and polyfluoroalkyl substances (PFAS) and polyfluorooctanoic acid (PFOA) pose significant risks to human health because they are both toxic and recalcitrant in the environment. Cadet Jackson Harris (US Air Force Academy) discussed techniques to isolate microorganisms capable of degrading these emerging contaminants through a laboratory incubation study where PFAS and PFOA were added over a six-week period. After observing a shift in the microbial communities due to the addition of the contaminants, bacteria and fungi capable of degrading PFAS were isolated to determine the classes of microorganisms and enzymes to optimize PFAS degradation at contaminated military sites. Cadet Harris, a member of the United States Air Force Academy’s International Genetically Engineered Machine team, helped to develop a technology to use a genetically engineered *Rhodococcus jostii* strain RHA1 to detect PFAS in laboratory media as a proof-of concept [26]. A novel detection technology will improve the ability to detect recalcitrant emerging contaminants in the environment.

Environmental microorganisms can be detrimental to military equipment by corroding and deteriorating the coatings on aircraft and vehicles because they harbor hydrolase enzymes capable of degrading polymer coatings [27]. For instance, *Papiliotrema laurentii* is a fungus that is capable of degrading a polyurethane coating [28]. Dr. Vanessa Varaljay (Air Force Research Laboratory) discussed bacteria and fungi present on the surfaces of the aircraft and trucks using metagenomics and metatranscriptomics approaches. Results from this study identified hundreds of putative hydrolase enzymes which are known to contribute to polyurethane polymer degradation. The information can be used to bioprospect for enzymes involved in bioremediation and to better understand biodegradation potential of microbial communities that are present on the polyurethane polymer surfaces.

### Microbiome analysis and discovery

Critical to microbiome research are the advancements in instrumentation, techniques, and analysis that enable visualization of microscopic organisms. An invited talk and three topical presentations discussed cutting-edge metagenomics and proteomics techniques used in environmental and human microbiome analysis and discovery. Prof. Pieter Dorrestein (University of California San Diego) opened the session by discussing the development of mass spectrometry approaches for microbiome data analysis of complex biological systems, which includes a discovery workflow [29]. Prof. Dorrestein has developed mass spectrometry approaches to estimate the false discovery rate for untargeted mass spectrometry [30] and computational tools linking genomics to spectra to mine for natural microbial products [31]. These tools allow mass spectrometry spectra to be ‘searched’ much like nucleotide and protein sequences are matched to a common database [32]. Using these tools, untargeted LCMS can be used to determine how the microbiome impacts the chemistry of the host. For example, differences in metabolites from conventionally-raised and germ-free mice fed the same diet were observed along the GI tract, and in different and distant organs, such as the brain. In a mouse study, Prof. Dorrestein described how repeated sleep disruption lead to vigilance and alertness deficits associated with a persistent shift in the fecal microbiome and metabolome [33], that could be rescued with dietary prebiotics [34]. He and his team developed a web-based portal called the Global Natural Products Social Molecular Networking [35] to share mass spectrometry data. Using these tools even individual diets can be assessed and predicted. More recently, these tools have been applied to research on natural products in the human gut [36] and microbial evolution to use organofluorine compounds [37].

Dr. Matthew Perisin, US Army Combat Capabilities Development Command—Army Research Laboratory, discussed challenges associated with microbial corrosion on Army equipment, which results in $20 billion dollars of damage each year. Microorganisms, regulated by nutrient access levels [38], colonize equipment surfaces, including metal, form biofilms, and cause general corrosion or pitting [39]. In studying the microbiome and using advanced techniques to analyze the microbiome, Dr. Perisin sought to establish controls to prevent corrosion by first sampling Army equipment and combining molecular analysis of the sample with culturing techniques. The focus of the remainder of the session was on the human microbiome, recognizing the need to understand the impact of the microbiome on human health and wellness to develop novel strategies to improve human performance.

Dr. Sophie Colston, US Naval Research Laboratory, investigated the impact of diving on the gut microbiome of US Navy divers in an effort to improve warfighter performance during complex missions. Ongoing projects are determining the effect of hypo- and hyperbaric conditions on the gut microbiome by investigating fecal microbiome diversity and metabolomics.

Dr. Ritesh Kumar (DuPont Nutrition and Biosciences), discussed efforts to characterize over 5,000 strains of bacteria from lean individuals to discover new probiotics to combat obesity. Using a mouse model, Dr. Kumar determined that the addition of four bacterial species improved insulin, glucose, and leptin levels. These studies highlight how advancement in microbiome analysis in discovery improves our ability to understand the role of the microbiome in human and environmental health.

### Lightning talks

#### Session #1

In this session, the themes centered on unique microbial properties including cold and soil adaptations and plastics degradation potential. The first talk from Dr. Robyn Barbato (ERDC-CRREL) focused on a new environmental intelligence platform called DRTSPORE, which predicts soil respiration activity across landscapes with respect to moisture and temperature. DRTSPORE was modeled on heterogeneous soil types, preliminarily tested on landscapes near CRREL with promising results, and will soon be validated and applied to arctic environments. Alison Thurston (ERDC-CRREL) presented on microbial changes based on 16S rRNA sequencing in Alaskan permafrost samples before and after thawing in the laboratory. Microbial communities were differentiated by sample site before thawing but interestingly microbial communities were not as distinctly clustered by sample site after thawing. Whether or not thawing is the driver in these results is next being explored. Flora Cullen (ERDC-CRREL) described a unique and novel psychrophile (growth < 20 °C) and psychrotroph (growth < 10 °C and > 20 °C) library of organisms collected from Toolik Lake, Permafrost Tunnel, and Greenland. So far, 68 organisms have been preserved and two of which showed growth at − 5 °C. Several organisms from the library produced pigments which can potentially be leveraged for coating materials for protection against extreme UV-irradiation as presented by Elizabeth Corriveau (ERDC-CRREL). Several of their genomes were sequenced using the Oxford Nanopore Technologies MinION and genes involved in pigmentation, such as in carotenoid production, were identified. Dr. Angelina Angelova (Naval Research Laboratory) showed with metagenomics sequencing from marine environments that similar microbial biofilm communities reside on different plastics regardless of their chemistry or composition. While genetic composition and metabolic potential were not significantly different between plastic types, many plastic-degrading enzymes were identified such as hydrolases and monooxygenases, demonstrating that these communities likely have general plastic-degrading potential.

#### Session #2

Lightning Talk Session #2 focused on a variety of studies that relate to the effects of dietary and environmental inputs on the activity and outcome of the human microbiome. Dr. Lisa Brenner (Rocky Mountain MIRECC) who has interests in understanding the mechanisms underlying posttraumatic stress disorder (PTSD) and mild traumatic brain injury (mTBI), has tested the hypothesis that these conditions are in part the result of chronic inflammatory disorders caused by reduced exposure to the microbial environment. When individuals afflicted with persistent PTSD and mTBI were administered a probiotic (*Lactobacillus reuteri*), significant decreases in C-reactive protein were observed versus controls as well as improvements in stress response during the challenging Trier Social Stress Test. As radiation exposure also elevates the inflammatory response and is thought to similarly modulate the composition and activity of the gut microbiome, Dr. Nabarun Chakraborty (Walter Reed Army Institute of Research) demonstrated that the microbiome composition of mice exposed to radiation was marked by an increase in alpha-diversity, an increase in the ratio of Firmicutes to Bacteroidates, and a beta-diversity reflecting a reduction in evenness over time. Also, PCA analysis of metabolomic panels show a time-dependent variation post-exposure to radiation. Toward a better understanding of the microbial ecology in the gut, Dr. Karley Mahalak (USDA – Agricultural Research Service) presented her group’s engineered in vitro reactor systems. This reactor is designed to mimic and control the conditions known to be present in the gut and provide a platform for comparative microbiome studies where input conditions, such as changes in diet and the introduction of antimicrobials, are varied. USDA is collaborating with the Army’s DEVCOM SC toward understanding the effects of nutritional supplements such polyphenols and dietary fibers on gut microbial activity toward the development of food supplement bars that can enhance gut and cognitive health for the Warfighter. In a separate study from the Army DEVCOM SC utilizing in vitro fermentation, Dr. Ida Pantoja-Feliciano (CCDC-SC) examined the effects of a sudden change from a habitual diet (HAB) to 21 days of a diet consisting of Meal, Ready-to-Eat (MRE) U.S. military combat ration on the population and functional dynamics of the human gut microbiome. With resistant starch as a fermenter substrate, there was no discernable impact on community dynamics when comparing fermentation seeded with fecal samples from MRE vs HAB diets; however, there was a slight increase in microbial species associated with short chain fatty acid production and glycoside hydrolase activity when the fermenter contained fecal samples from an MRE diet (vs HAB diet). Dr. Jordan Zambrana (EPA – Indoor Environments Division) discussed his Agency’s strategies for improving indoor air quality (IAQ) to protect human health with an appreciation for how the link between indoor air chemistry and the indoor microbiome affects IAQ. Given this link between the chemistry and microbiology, discussion focused on outstanding scientific questions of interests to the EPA related to an understanding of indoor microbial ecologies, the nutrients available to sustain the indoor microbiome and a better understanding of how environmental chemicals in turn affect human physiology including the human microbiome.

## Conclusions

The 4th Annual TSMC Symposium highlighted that microbiome research is supported by a vibrant and diverse DoD research community and, more importantly, DoD leadership. The research presented described utilizing microbiomes to improve warfighter health and performance, maintaining warfighter systems and materiel, and monitoring and predicting environmental conditions and resiliency to perturbation. Research areas spanned from fundamental characterization to actionable predictive outcomes and interventions. Pivoting away from a ‘one size fits all’ approach was a recurrent theme, and modulation of microbiomes using natural and engineered systems was a clear research direction. What was also clear was that microbiome research requires multidisciplinary teams so that resources, expertise, and data could be leveraged and shared to maximize the beneficial effects of the microbiomes that were discussed. To that end, discussions around how data could be shared and leveraged between studies came to the fore. These focused on how large data sets could be shared and how existing computational resources could be accessed. Concomitant with this notion were discussions on how to standardize studies to facilitate between study comparisons. It was acknowledged that the standards may change as the field develops, and that these standards may be context dependent (i.e., the standards may be different for therapeutic outcomes vs. monitoring outcomes) but it was agreed that a general framework to follow for all DoD microbiome research data collection and analysis would be beneficial. These points were emphasized as a key part of the DoD Biotechnology Modernization pillar. Finally, gaps in DoD microbiome research were discussed focusing in on human microbiomes other than the gut. While DoD funds some research into non-human gut microbiomes (such as otic, skin, nasal, and lung), it is not a DoD service laboratory focus currently. The meeting highlighted that, as in microbiomes themselves, ‘it takes a village’, and that sharing capabilities and expertise, and, perhaps most importantly, continually communicating the latest advances to avoid redundancy and maximize resources will remain critical to realizing the full impact of microbiome-based solutions to advance DoD interests.

## Data Availability

Not applicable.

## Abbreviations

AFRL: Air Force Research Laboratory
ARL: Army Research Laboratory
DEVCOM: Combat Capabilities Development Command
CFD: Combat Feeding Directorate
CRREL: Cold Regions Research and Engineering Laboratory
DoD: US Department of Defense
EPA: Environmental Protection Agency
ERDC: US Army Engineer Research and Development Center
LANL: Los Alamos National Laboratory
LL: Lincoln Laboratory
MIRECC: Mental Illness Research Education and Clinical Center
MIT: Massachusetts Institute of Technology
NRL: Naval Research Laboratory
SC: Soldier Center
TSMC: Tri-Service Microbiome Consortium
UCSD: University of California, San Diego
UMASS: University of Massachusetts
USAFA: US Air Force Academy
USDA-ARS: US Department of Agriculture – Agricultural Research Service
VA: Department of Veterans Affairs
WRAIR: Walter Reed Army Institute of Research

## Declarations

### Disclaimer

All opinions or assertions contained herein are the private views of the authors and are not to be construed as official or reflecting the views of the Army, Navy, Air Force, or the Department of Defense. Any citations of commercial organizations and trade names in this report do not constitute an official Army, Navy, Air Force, or Department of the Defense endorsement or approval of the products or services of these organizations. Approved for public release; distribution is unlimited.
